# Transcriptome profiles of three Muscat table grape cultivars to dissect the mechanism of terpene biosynthesis

**DOI:** 10.1038/s41597-019-0101-y

**Published:** 2019-06-13

**Authors:** Lei Sun, Baoqing Zhu, Xuanyin Zhang, Guojun Zhang, Ailing Yan, Huiling Wang, Xiaoyue Wang, Haiying Xu

**Affiliations:** 1 Beijing Academy of Forestry and Pomology Sciences, Beijing, 100093 China; 2 College of Biological Sciences and Technology, Beijing Forestry University, Beijing, 100083 China; 3 Key Laboratory of Biology and Genetic Improvement of Horticultural Crops (North China), Ministry of Agriculture and Rural Affairs, Beijing, 100093 China; 4 Beijing Engineering Research Centre for Deciduous Fruit Trees, Beijing, 100093 China

**Keywords:** Plant molecular biology, Secondary metabolism, RNA sequencing

## Abstract

*Vitis vinifera* is widely grown worldwide for making wine and for use as table grapes. Of the existing cultivars, some have a floral and fruity flavour, referred to as a Muscat flavour. It is well-documented that this flavour originates from a series of terpene compounds, but the mechanism of terpene content differences among the Muscat-type cultivars remains unclear. Transcript and terpene metabolite profiles were integrated to elucidate the molecular mechanism of this phenomenon. In this research, three genotypes with different aromatic strengths were investigated by RNA sequencing. A total of 27 fruit samples from three biological replicates were sequenced on Illumina HiSeq2000 at three stages, corresponding to the veraison; berries had intermediate Brix value and were harvest-ripe. After quality assessment and data clearance, a total of 254.18 Gb of data with more than 97% Q20 bases were obtained, approximately 9.41 Gb data were generated per sample. These results will provide a valuable dataset for the discovery of the mechanism of terpene biosynthesis.

## Abstract (summary)

**Table:** 

Design Type(s)	transcription profiling design • gene expression analysis objective
Measurement Type(s)	transcription profiling assay
Technology Type(s)	RNA sequencing
Factor Type(s)	cultivar • biological replicate • developmental stage
Sample Characteristic(s)	Vitis vinifera • berry

Machine-accessible metadata file describing the reported data (ISA-Tab format)

## Background & Summary

The trait of aroma is one of the most important parameters for the quality of grapes and is the main concern when consumers buy grape products. For genetic improvement research and breeding, the biosynthesis mechanism of aromatic compounds and their regulation has attracted much attention. Terpenes are the typical aromatic compounds in Muscat grapes, and they belong to the second metabolites^1–4^; they have a low olfactory threshold and can be easily precepted by humans. The terpenes mainly exist in the pericarp and in the flesh of some cultivars^5^, with their content being affected by the genotype^6,7^, developmental stage^8,9^, environment and management of the grape^10–13^. Terpenes have two forms: the free form, which directly leads to the aromatic flavour, and the glycoside bound form, in which the potential aromatic compounds transfer to the free form by hydrolysis^14–16^.

Biologically, the biosynthesis of terpene compounds in plants are synthesized by two pathways, the methyl-erythritol-4-phosphate pathway (DXP/MEP) in the plastid and the mevalonate pathway (MVA) in the cytoplasm^17^, with terpenes located in the mesocarp and pericarp^18^. Starting from pyruvic acid and 3-phosphate glyceraldehyde, by 1-deoxy-D-xylulose-5-phosphate synthase (DXS), which is the entrance enzyme in the MEP pathway, the two compounds were changed into 1-deoxy-D-xyulose-5-phosphate and, then, through six enzymatic reactions, were converted into geranyl-diphosphate (GPP). Geranyl-diphosphate was the substrate for all the terpenes. Then, by a series of terpene synthases, the GPP was synthesized into hemiterpenes (C5), monoterpenes (C10), sesquiterpenes (C15) or diterpenes (C20)^19–22^.

The genetic mechanism of Muscat flavour in grapevines has been studied through quantitative trait loci analysis (QTL) in different F1 populations^23,24^, and in selfing populations, it has been shown that VvDXS is a structural candidate gene for geraniol, nerol, and linalool concentrations in wine grapes^25^. Battilana reported that single nucleotide polymorphism (SNP) mutations in VvDXS are the main cause of the Muscat flavour. The substitution of a lysine with an asparagine at position 284 of the VvDXS amino acid sequence affects the monoterpene content of Muscat flavour and neutral cultivars^26^.

In Muscat grapes, some cultivars have a very strong flavour, while others have moderate or light flavour. The terpene type and concentration varied among the cultivars. To date, terpene accumulation has been well-documented in some wine grapes. Terpene accumulation in developing Gewurztraminer grapes has been shown to be correlated with an increase in the transcript abundances of early terpenoid pathway enzymes^27^. Some transcription factors involved in controlling terpene biosynthesis have been predicted in the grapevine cultivar Muscat Blanc à Petits Grains through gene co-expression network analysis^28^. A Nudix hydrolase was also found to be a component of a terpene synthase-independent pathway, with cytochrome P450 hydroxylases, epoxide hydrolases and glucosyltransferases genes potentially involved in monoterpene metabolism^29^. However, there are few reports on the table grape^30^.

In this study, we present the transcriptome analysis of three genotypes of table grapes. During berry development, 27 samples, in total, were sequenced on the Illumina HiSeq Platform. After quality assessment and data clearance, a total of 254.18 Gb high-quality base pairs with more than 97% Q20 bases were obtained, and an approximately 9.41 Gb per sample. In the aggregate, a total of 776 million reads were yielded, with an average of 31.66 million reads per sample. Furthermore, approximately 76.65% of the total reads were uniquely aligned to the grape genome (V2)^31^. These data will provide useful information for investigating terpene biosynthesis.

## Methods

### Overview of the experimental design

**Fig. 1:**
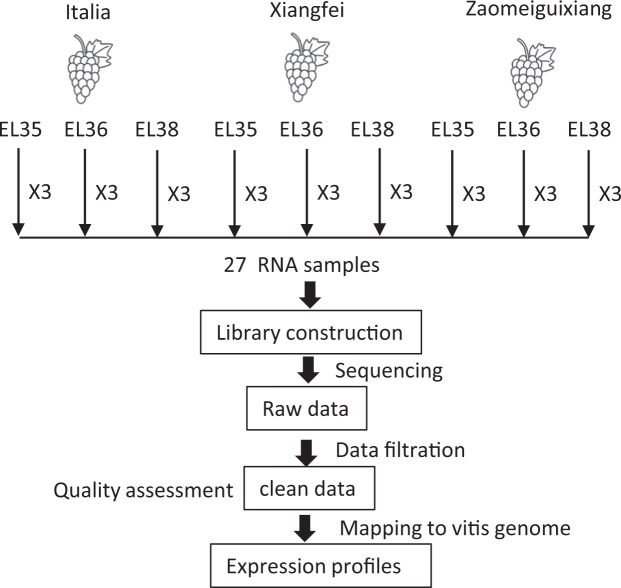
Flowchart of the experimental design. Berry samples were collected at three developmental stages, and three biological replicates per sample were used for transcriptome sequencing. All raw reads were quality controlled and assessed. Then, the clean data were mapped to the *V. vinifera* reference genome (V2) by Hisat2. Gene expression levels were calculated with RSEM.

The berries of three genotypes were collected at three developmental stages. Approximately 300 grape berries were randomly collected for each replicate, with three replicates harvested for each stage. The experimental design and analysis pipeline are shown in Fig. 1.

### Materials and methods

#### Plant materials

Three *V. vinifera* cultivars were used for transcript study. ‘Xiangfei’ was registered by our team and has a strong Muscat flavour and a green to golden skin colour, while ‘Italia,’ the famous mid-late season table grape cultivar that originated in Italy, has a moderate Muscat flavour. ‘Zaomeiguixiang’ has a purple-reddish colour and a strong Muscat flavour.

#### Sampling

The vines were grown in the experimental vineyard at the Beijing Academy of Forestry and Pomology Sciences in China (39°58′N and 116°13′E) under a plastic cover and were trained into a two-wire vertical trellis system with a 2.5-m row space and a 0.75 m plant space. In 2017, berry samples from three vines were harvested at the developmental stages corresponding to EL35, EL36, and EL38^32^. The berry begins to colour and soften at EL 35 (about 5% of the berries started to colour and soften), progresses to the complete veraison with an intermediate Brix of EL 36, and reaches harvest ripeness at EL38. At each stage, three replicates were harvested; approximately 300 grape berries were randomly collected for each replicate.

#### Physiochemical parameters

Fifty berries of each replicate were pressed and centrifuged to determine total soluble solids (TSS), pH value and titratable acidity. TSS was measured by a digital refractometer (PAL-1, Atago, Tokyo, Japan). The pH value was measured by a pH meter (FiveGo F2-Standard, Mettler Toledo, Switzerland). Titratable acidity was analysed by titration with NaOH (0.1M) to the end point of pH 8.2 and expressed as tartaric acid equivalents in accordance with the National Standard of People’s Republic of China (GB/T15038-2006, 2006). The other berries were then frozen in liquid nitrogen and stored at −80 °C.

#### RNA extraction and sequencing

The extraction of total RNA from the berries was carried out by a Plant RNA extraction kit (Aidlab Biotechnologies, Beijing, China). The quality of the RNA was verified by agarose gel electrophoresis, and the concentration was determined by the absorbance ratio (A260/A280, 1.8–2.0) on an Implen P330 nanophotometer (Implen GmbH, Munich, Germany).

The RNA-Seq libraries were constructed from 27 samples according to the methods of Wang^33^. The enriched mRNA was obtained by using oligo (dT) magnetic beads then fragmented by 94 °C for 5 min. cDNA was synthesized by Superscript®III Reverse Transcriptase, followed by purification, end repair and dA-tailing and was then ligated with the sequencing adaptor. Afterwards, PCR amplification was conducted by indexed primers. The constructed library was QC checked by Agilent 2100 Bioanalyzer and ABI StepOnePlus Real-Time PCR System and then sequenced by Illumina HiSeq2000 platform at BGI Life Tech Co., Ltd. (Shenzhen, China). Low quality reads (more than 20% of the base qualities are lower than 10), reads with adaptors and reads with unknown bases (N bases more than 5%) were filtered to get clean reads and were stored in FASTQ format. The clean reads were mapped onto the reference grapevine genome (V2) using Hisat2^34^.

## Data Records

The RNA-Seq clean data of the 27 samples were deposited at the NCBI Sequence Read Archive with accessions SRP184152^35^. The files of gene expression level were deposited in NCBI’s Gene Expression Omnibus (GEO), and are accessible through GEO Series accession number GSE130386^36^. The information of the differentially expressed genes (DEGs) between samples were deposited in figshare^37^.

## Technical Validation

### Quality control

The physiochemical parameter of the samples was shown in Table 1. A total of 27 RNA samples were prepared and sequenced, with the sequencing depth ranging between 22.48 and 33.08 million reads; the Q20 values for the clean reads were above 97%, and the average mapping ratio was 84.72% (Online-only Table 1).

**Table 1:** Physiochemical parameters for each sample.

Sample name	Total soluble solids	Titratable acidity(g/l)	pH
X-EL35-1	10.84	4.25	3.11
X-EL35-2	10.80	4.20	3.15
X-EL35-3	10.95	4.26	3.16
X-EL36-1	13.46	4.01	3.53
X-EL36-2	13.30	3.98	3.50
X-EL36-3	13.80	4.05	3.58
X-EL38-1	16.62	3.73	3.75
X-EL38-2	16.40	3.70	3.71
X-EL38-3	16.42	3.68	3.77
Y-EL35-1	5.18	5.15	3.07
Y-EL35-2	5.20	5.20	3.05
Y-EL35-3	5.18	5.22	3.01
Y-EL36-1	7.61	4.85	3.14
Y-EL36-2	7.45	4.80	3.18
Y-EL36-3	7.40	4.79	3.17
Y-EL38-1	14.80	4.51	3.47
Y-EL38-2	14.50	4.52	3.48
Y-EL38-3	14.57	4.48	3.45
Z-EL35-1	9.78	3.96	3.30
Z-EL35-2	9.70	3.95	3.32
Z-EL35-3	9.80	3.99	3.32
Z-EL36-1	12.90	3.48	3.75
Z-EL36-2	12.95	3.55	3.78
Z-EL36-3	12.88	3.52	3.71
Z-EL38-1	17.25	3.05	3.85
Z-EL38-2	17.20	2.96	3.80
Z-EL38-3	17.29	3.07	3.82

X stands for cultivar Xiangfei, Y for cultivar Italia and Z for cultivar Zaomeiguixiang. EL35: the berry begins to colour and soften, EL36: complete of veraison with an intermediate Brix, EL38: berry reaches harvest ripeness.

### Analysis of RNA-Seq data

**Fig. 2:**
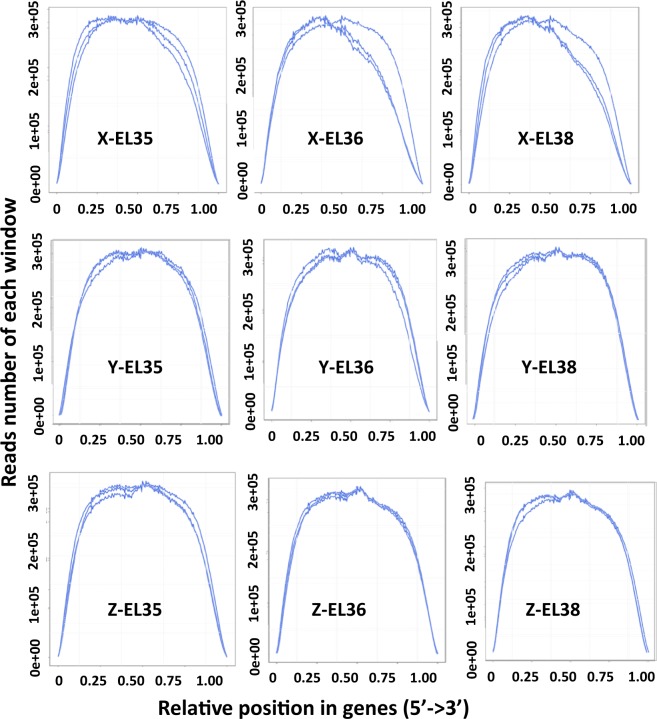
Reads distribution on transcripts. The x-axis represents the position along transcripts, and the y-axis represents the number of reads.

**Fig. 3:**
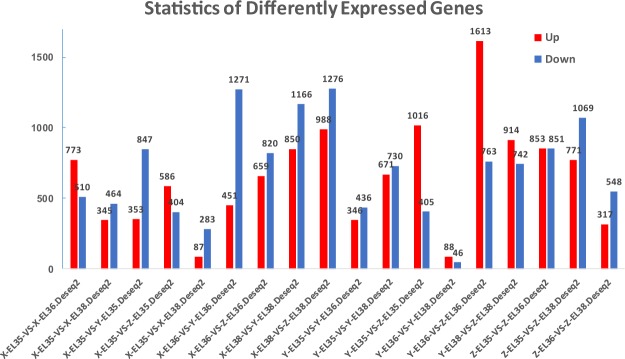
Statistics of differently expressed genes. The X-axis represents the comparison method between groups and the y-axis represents DEG numbers. The red colour represents upregulated DEGs, and the blue colour represents downregulated DEGs.

After novel transcript detection, novel coding transcripts were merged with reference transcripts to get a complete reference. Clean reads were mapped to the transcript by using Bowtie2^38^. Gene expression levels were calculated with RSEM^39^. The distribution of reads based on the detection of read coverage skewness showed good fragmentation randomness (Fig. 2). The differentially expressed genes (DEGs) between samples were identified by the R package, DESeq2^40^. The DEGs with a |log2ratio| ≥ 1 and a false discovery rate probability ≤ 0.001 were considered statistically significant. The statistical analyses of DEG are shown in Fig. 3.

## Usage Notes

The RNA-Seq fastq.gz files were deposited at Gene Expression Omnibus and can be downloaded using the fastq-dump tool of the SRA Toolkit (https://www.ncbi.nlm.nih.gov). The V2 reference genome of *V. vinifera*, the annotated file, could be retrieved at (http://genomes.cribi.unipd.it/grape/).

### ISA-Tab metadata file

Download metadata file

## Data Availability

SOAPnuke: https://github.com/BGI-flexlab/SOAPnuke. Version: v1.5.2. Parameters: -l 5 -q 0.51 -n 0.55 -i -Q 2–seqType 1. HISAT2: http://www.ccb.jhu.edu/software/hisat. Version:v2.0.4.Parameters:–phred64–sensitive–no-discordant–no-mixed -I 1 -X 1000. Bowtie2: http://bowtie-bio.sourceforge.net/Bowtie2. Version: v2.2.5. Parameters: -q–phred64–sensitive–dpad 0–gbar 99999999–mp 1,1–np 1–score-min L,0, −0.1 -I 1 -X 1000–no-mixed–no-discordant -p 1 -k 200. RSEM: http://deweylab.biostat.wisc.edu/RSEM. Version: v1.2.12. Parameters: default.

## Online-only Table

**Online-only Table 1:** Statistics of sequencing data for each sample.

Sample	Total Raw Reads(Mb)	Total Clean Reads(Mb)	Total Clean Bases(Gb)	Clean Reads Q20(%)	Total Mapping Ratio%	Uniquely MappingRatio%	Accession number (Biosample)
X-EL35-1	32.79	30.31	9.78	98.75	82.79	76.97	SAMN10880015
X-EL35-2	32.67	30.17	9.69	98.82	80.71	72.02	SAMN10880016
X-EL35-3	32.58	30.04	9.70	98.68	81.33	76.37	SAMN10880017
X-EL36-1	32.28	29.26	9.59	98.34	85.30	76.21	SAMN10880018
X-EL36-2	31.96	28.86	9.50	98.61	94.53	79.67	SAMN10880019
X-EL36-3	30.29	27.61	8.96	98.73	91.97	80.36	SAMN10880020
X-EL38-1	32.05	29.53	9.50	98.79	79.21	73.29	SAMN10880021
X-EL38-2	31.58	29.07	9.38	98.79	85.37	77.24	SAMN10880022
X-EL38-3	32.46	29.62	9.63	98.26	89.61	81.99	SAMN10880023
Y-EL35-1	32.70	30.22	9.74	98.74	71.65	68.36	SAMN10880024
Y-EL35-2	32.63	29.85	9.71	98.66	70.39	64.96	SAMN10880025
Y-EL35-3	33.08	30.73	9.86	98.80	82.73	76.09	SAMN10880026
Y-EL36-1	32.71	29.77	9.72	98.68	83.51	70.43	SAMN10880027
Y-EL36-2	32.89	30.02	9.78	98.67	84.90	75.87	SAMN10880028
Y-EL36-3	32.54	29.09	9.69	98.48	82.47	74.05	SAMN10880029
Y-EL38-1	32.37	29.26	9.62	98.18	66.95	58.86	SAMN10880030
Y-EL38-2	32.98	30.29	9.82	98.71	82.08	74.71	SAMN10880031
Y-EL38-3	31.89	27.00	9.50	97.75	83.90	73.87	SAMN10880032
Z-EL35-1	33.01	30.53	9.83	98.76	94.94	87.08	SAMN10880033
Z-EL35-2	28.17	24.35	8.38	97.97	80.48	71.95	SAMN10880034
Z-EL35-3	32.90	30.23	9.78	98.66	94.43	87.15	SAMN10880035
Z-EL36-1	32.24	29.09	9.58	98.30	86.69	79.18	SAMN10880036
Z-EL36-2	32.68	29.88	9.71	98.59	93.01	84.9	SAMN10880037
Z-EL36-3	32.81	29.80	9.75	98.56	90.54	82.82	SAMN10880038
Z-EL38-1	25.26	22.06	7.50	98.05	89.97	82.28	SAMN10880039
Z-EL38-2	32.92	30.20	9.78	98.65	88.41	82.38	SAMN10880040
Z-EL38-3	22.48	19.16	6.70	97.86	89.56	80.46	SAMN10880041

X stands for Xiangfei, Y for Italia and Z for Zaomeiguixiang, Total Raw Reads (Mb): The reads amount before filtering, Unit: Mb. Total Clean Reads (Mb): The reads amount after filtering, Unit: Mb. Total Clean Bases (Gb): The total base amount after filtering, Unit: Gb. Clean Reads Q20(%): The Q20 value for the clean reads. Total Mapping Ratio: The percentage of mapped reads. Uniquely Mapping Ratio: The percentage of reads that map to only one location of reference. EL35: the berry begins to colour and soften, EL36: complete of veraison with an intermediate Brix, EL38: berry reaches harvest ripeness.
